# A Tribute to Robert (Bob) Sim—Personal Memories of Working in Bob’s Lab

**DOI:** 10.3390/v13091696

**Published:** 2021-08-26

**Authors:** Samantha Williams, Alexander Steinkasserer

**Affiliations:** 1 Trinity Research & Innovation, O’Reilly Institute, Trinity College Dublin, Dublin 2, Ireland; 2 Department of Immune Modulation, University Hospital Erlangen, Friedrich-Alexander Universität Erlangen-Nürnberg, Hartmannstrasse 14, D-91052 Erlangen, Germany

**Keywords:** Robert B. Sim, obituary, complement

## Abstract

This article is intended as a tribute to Robert B. Sim through the sharing of personal memories and anecdotes from two of Bob’s lab members who worked in his lab between 1989 to 1994.

## 1. Personal Memories from Samantha Williams, D. Phil. Student, 1990 to 1994

I was privileged to join Bob’s lab within the MRC Immunochemisty Unit in 1990 to carry out an MRC-funded D.PhiI. studentship. I had written to Bob after completing my BSc in Biochemistry (at the University of Wales, College Cardiff) looking for a place to carry out a PhD. I was a little ‘green’ to say the least, on what was involved in carrying out a D.Phil. but I had very much enjoyed learning about the complement system during my B.Sc., and I knew that Bob was a leader in the field. This was in the days before internet, e-mails, and mobile phones, but in corresponding with Bob (letters and phone calls!), and in him offering me a place in his lab, I had the feeling that the chance of being one of Bob’s students was a great opportunity for me. A part of me always felt that Bob took a bit of a risk on this unknown Welsh girl, but his uniquely inclusive manner and open mind was always apparent in the way that he approached everything in his lab. 

I realise now that I was among the first few of Bob’s D.Phil. students, but it felt right from the start as if Bob had been supervising for a lifetime as he was such a natural teacher. Bob gave me the domain structure of factor B of the alternative pathway of the complement system to work on. Factor B research might have been considered as somewhat traditional and not as ‘hot’ as some of the other areas of complement-related research being explored at the time. However, Bob had designed a great project for me which allowed me to expose and explore the gaps in our knowledge of studying factor B. It was an exciting project that was individual and original, and gave me experience and training in a wide variety of techniques, leading, towards its completion, to a successful collaboration with Bob’s collaborator Steve Perkins of the Royal Free Hospital School of Medicine in London. 

Bob was a tremendously dedicated supervisor and had a huge amount of patience. These attributes were needed in spades as, at the start, my technical abilities were very limited. Luckily for me, Bob’s approach was to concentrate on basic practical skills, and to write out in a great amount of detail step by step instructions on experiments for me to try out. Very slowly, I began to build up the confidence and the essential skills needed to carry out basic complement and protein chemistry experiments. My arrival in Oxford was exciting and, in some ways, daunting, with the traditions of living in an Oxford College to become accustomed to. There is no doubt that Bob’s consistent, supportive, structured, and diligent approach to supervising was a great aid to me in settling in and to improving my potential as a young research scientist, as well as a person. 

Bob did not shy away from direct guidance at certain key times. I can clearly remember him letting me know that I should focus a little less on my social life and instead knuckle down to achieving some results to present at my first conference. This clear direction worked a charm, as, like all of Bob’s lab members, I was very keen to be someone that Bob was proud of. 

As with my experiments, Bob took great care to provide detailed feedback on my writing, from drafting my first abstract for a conference, to later publications and my D.Phil. thesis [1,2,3,4,5,6]. At first, anything I wrote came back with so many written changes from Bob, it was almost unrecognisable. I remember a very clearly written note in the margin of a piece of written work stating “*Bull…*!” I was appalled at the time that I had written something so inaccurate. However, memories shared recently with my co-author here, Professor Alexander Steinkasserer, a fellow lab member at the time, have highlighted that I was probably not the only one to receive this type of feedback!

Gradually, I began to improve, both technically and in my writing, but only as a direct result of Bob’s attention to detail, and honest, clear guidance. I remember so clearly the day that Bob stated that I was “becoming technically skilled” and likewise the day an abstract was returned from him without a single change and just a “very well written” at the end of the abstract. The fact that this praise meant so much to me at the time and that those memories are still clearly with me over thirty years later are testaments to Bob and his strengths as a scientist, a supervisor, and most importantly as a person.

**Figure 1:**
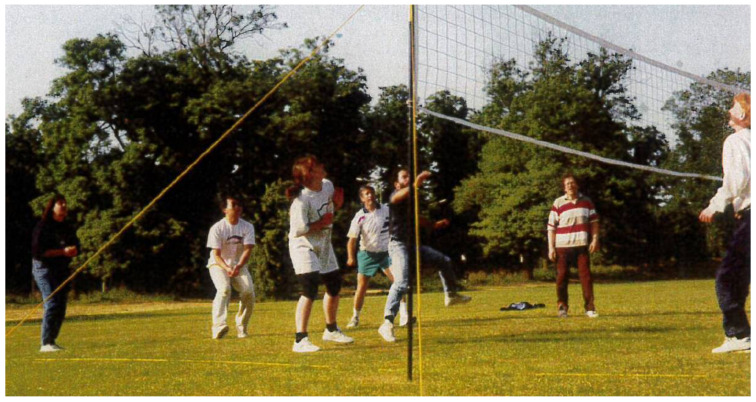
Bob and some of his lab members at an MRC Immunochemistry Unit volleyball event, Oxford, circa 1992.

Although Bob spoke gently and was always calm, this was a subtle counter to his very sociable nature (Figure 1) and his great sense of humour. It was rare that his very small office in the lab was ever empty, if the door was left ajar. Callers from around the MRC Immunochemistry Unit dropped in and, although there were certainly many serious scientific discussions, there was also consistent laughter, general chat, and—in those days—cigar smoke. Bob had a habit of greeting people in the lab with a simple “Hi” with their name at different parts of the day, and these behaviours were so typical of Bob. They provide just one example of the courtesy and warmth Bob gave to everyone. 

Bob was very generous, and regularly took us out for lunch as a lab, and Friday evening visits to the Royal Oak were a part of the lab’s schedule through the year. Bob and Edith were the kindest of hosts at social gatherings for lab members at their home, and I remember well their children Grace and Francis being very little and great fun at those gatherings.

Bob was always keen to look out for my welfare during my time in his lab, and I know this was the case with all of his lab members. From difficulties with accommodation, to a potential new employer being slow in making a decision, he offered his time and his attentive and caring ear, coupled with the offer of help in any way that he could. 

**Figure 2:**
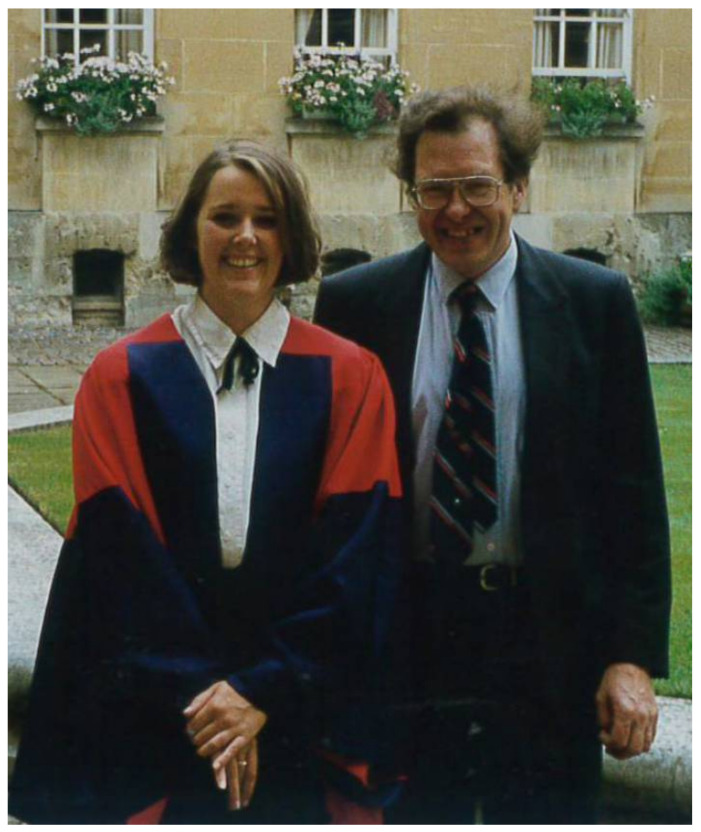
D.Phil. Graduation Day, with Bob, July 1994, Trinity College Oxford.

My D.Phil. Graduation Day (Figure 2) was a really memorable day as I was delighted that Bob and Edith were able to join our celebrations and could meet my Welsh family and friends.

After leaving Bob’s lab in 1994, I worked in industry as a research scientist before changing career direction to join the MRC’s Technology Transfer Group. One of the best things about my new job in London was getting the chance to visit the MRC Immunochemistry Unit in my new role and to meet Bob once again. Bob was extremely supportive to me, both in Oxford and in my continuing career. It struck me how knowledgeable he was about the role of the MRC group I started working with, and this was a great help to me in starting out. His continued support was greatly appreciated by me. 

After leaving the UK in 2000 to live in Dublin, Ireland, and continue my career in technology transfer, it was an honour to keep in touch with Bob every few years. He was always so interested in what I was doing, as well as being totally up to date and full of news of everyone that had been in the lab at the same time as me. It was always a pleasure to hear about Bob’s life, and news of Edith, Grace, and Francis.

Bob was an extraordinary scientist and an extraordinary teacher, a wonderful role model, and a true gentleman. He has had a significant and lasting influence on my career, as well as my life, and it was a privilege to have known him. There is no doubt that he will be sorely missed and that he leaves a positive mark on us all.

## 2. Personal Memories from Alexander Steinkasserer, Postdoctoral Fellow, 1989 to 1993

In September 1989, I had the great opportunity to move from Munich to Bob’s lab in Oxford. I must emphasise right from the beginning what a fantastic experience this was, and the best decision I could have made. Already during my job interview, a few months prior to me joining Bob’s team, I was struck by his gentle and simultaneously impressive personality. I was also fascinated by his extremely interesting way of describing and explaining his scientific projects and areas of interest, and what role I could play here. Bob made me feel at home from day one, and I was convinced that working with him and his team would present an extremely important and decisive turning point in my life, not only for my scientific career. I was then also fortunate enough to be granted an “Oxford Centre for Molecular Sciences” (OCMS) research fellowship, to support my work in Bob’s research team. 

I still vividly remember, as if it was yesterday, how I embarked the ferry at Calais, leaving the continent behind me and looking ahead at the impressive white cliffs of Dover. Driving on the “wrong” side of the road (for me), was initially a slight challenge, however, knowing that I would soon join Bob’s lab group lightened my journey. Bob kindly booked a room for me in the nearby Halifax House for the first couple of weeks, easing my start, prior to my move to Islip, where I enjoyed my time living in the roof-top apartment of “The Old Rectory”.

**Figure 3:**
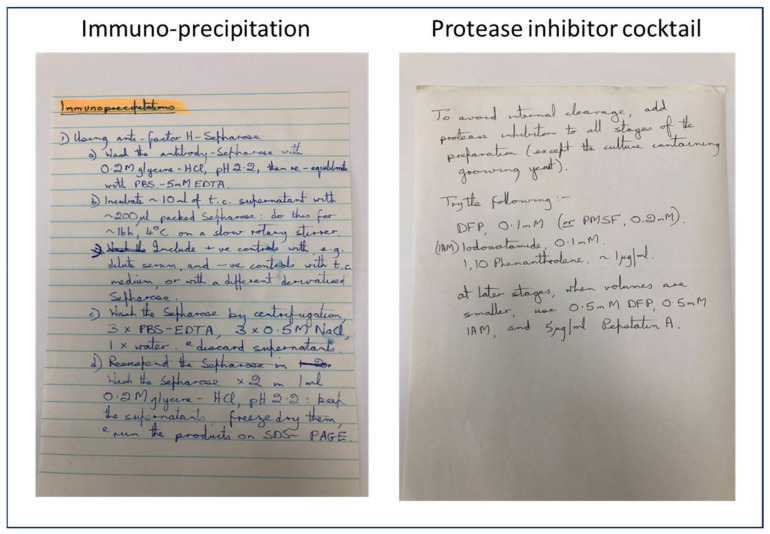
An example of Bob’s handwritten detailed methods.

The first days in Bob’s lab were a real eye-opener for me, as the atmosphere in his lab contrasted sharply with that which I had experienced in my former lab in Munich. Due to his caring and great personality, Bob created an extremely pleasant and at the same time very productive environment. The routine daily start in the lab was particularly congenial, and supported being able to pick up where you left off with the work the day before, without any unnecessary distractions. Working in Bob’s lab was a great experience! Whenever needed, and initially this was quite frequently the case, I could approach Bob in his nearby office, and he was always exceptionally helpful. For example, he would first patiently explain the experimental background and then write down the exact protocol details to be followed, whether it be for immuno-precipitation, or the composition of a protease inhibitor cocktail to avoid protein degradation (see Figure 3), or something else.

Bob gave me a very interesting initial project, which involved research work on factor H, a very important complement control protein composed of 20 individual structural modules, called complement control protein repeats (CCPs). Each of these CCPs share a common overall topology, and it was my job to recombinantly express, purify, concentrate, and characterize single or double modules. Subsequently, in cooperation with the NMR group of Prof. Ian D. Campbell, the solution structures were determined. Prior to my work in Bob’s lab, I had gained experience regarding the implementation of molecular biology techniques, however, I initially lacked the required experience in protein biochemistry and related techniques. I therefore benefitted greatly from Bob’s support, which was both needed and extremely helpful in successfully advancing this project. Finally, together with our collaborators from Ian’s lab, in particular Paul Barlow, this work turned out to be a great success and led to the publishing of two very interesting papers [7,8].

Bob was not only interested and highly knowledgeable regarding the complement system, but also regarding immunological and other scientific related fields. This gave me the opportunity to extend my project portfolio and to work on two additional molecules, namely the beta 2-glycoproten I (beta 2I), also composed of CCP modules, and the Interleukin-1 receptor antagonist (IL-1 RA). Once again, Bob was extremely supportive, and I sought his valuable advice on multiple occasions when I was at a loss as how to continue, or when the projects were not running as smoothly as they should have. Finally, these projects also progressed very well, the results of which were published, with both Bob and I as authors. The research work on our projects led to 14 publications [6,7,8,9,10,11,12,13,14,15,16,17,18,19] with Bob, evidence of the very productive time in his lab in Oxford, for which I am most grateful to him.

Bob was, both as a scientist and supervisor in the lab, but also on a personal and interactive level, extraordinary. It was definitely from him that I learned how to run a laboratory. I also learned from his example how to interact best with people, i.e., always in a friendly supportive manner, and how to motivate young as well as slightly “older” scientists, without putting obvious pressure on them. The latter was definitely not necessary, as everybody in his lab was very proud to be working there. We therefore had a particularly happy working environment, despite the long working hours. Friday evenings presented an exception, when we all met in the local pub and enjoyed taking about science or just chatting about other topics. When reflecting back on this time, I am also particularly thankful to Edith, as well as Grace and Francis, and for the memorable gatherings we enjoyed in your home. 

Even after I had left the MRC Unit in Oxford to take up my subsequent position in the pharmaceutical industry, and later, back in academia, as professor at the University Hospital in Erlangen, I continued to enjoy Bob’s valuable and helpful advice. He gladly took time for me when I called him from time to time to ask his advice, or he’d give me an update of how the former and present group members were doing. This always gave me the immediate feeling of being back in his office, just like “in the good old days”, once again sharing great moments together. 

In short, Bob was a most extraordinary person and a brilliant teacher and scientist! He influenced my thinking and my career profoundly, and although we miss him immensely, he will continue to live in our hearts and minds forever.
